# Ethyl 3-[7-eth­oxy-6-(4-meth­oxy­benzene­sulfonamido)-2*H*-indazol-2-yl]propano­ate

**DOI:** 10.1107/S1600536812007519

**Published:** 2012-03-03

**Authors:** Najat Abbassi, Bassou Oulemda, El Mostapha Rakib, Detlef Geffken, Hafid Zouihri

**Affiliations:** a Laboratoire de Chimie Organique et Analytique, Université Sultan Moulay Slimane, Faculté des Sciences et Techniques, Béni-Mellal, BP 523, Morocco; b Department of Pharmaceutical Chemistry, Institute of Pharmacy, University of Hamburg, Hamburg, Germany; c Laboratoires de Diffraction des Rayons X, Centre Nationale pour la Recherche Scientifique et Technique, Rabat, Morocco

## Abstract

In the title compound, C_21_H_25_N_3_O_6_S, the dihedral angle between the meth­oxy­benzene and indazole rings is 74.96 (5)°. The crystal packing is stabilized by an N—H⋯O hydrogen bond into a two-dimensional network. In addition, C—H⋯π inter­actions and a π–π contact, with a centroid–centroid distance of 3.5333 (6) Å, are observed. The crystal packing is stabilized by N—H⋯O and C—H⋯O hydrogen bonds.

## Related literature

For related structures, see: Abbassi *et al.* (2011*a* ▶,*b* ▶). For the biological activity of sulfonamides, see: Soledade *et al.* (2006 ▶); Lee & Lee (2002 ▶).
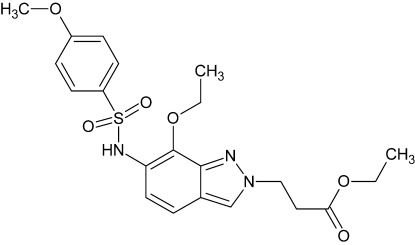


## Experimental

### 

#### Crystal data

C_21_H_25_N_3_O_6_S
*M* *_r_* = 447.50
Triclinic, 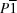

*a* = 9.1163 (4) Å
*b* = 10.9161 (5) Å
*c* = 11.2959 (5) Å
α = 77.259 (2)°
β = 77.364 (2)°
γ = 88.562 (2)°
*V* = 1069.55 (8) Å^3^
*Z* = 2
Mo *K*α radiation
μ = 0.20 mm^−1^
*T* = 296 K
0.32 × 0.31 × 0.19 mm

#### Data collection

Bruker APEXII CCD detector diffractometer
Absorption correction: multi-scan (*SADABS*; Sheldrick, 2003 ▶) *T*_min_ = 0.940, *T*_max_ = 0.964
21582 measured reflections
4187 independent reflections
3834 reflections with *I* > 2σ(*I*)
*R*_int_ = 0.025

#### Refinement

*R*[*F* ^2^ > 2σ(*F* ^2^)] = 0.030
*wR*(*F* ^2^) = 0.084
*S* = 1.06
4187 reflections
283 parameters
H-atom parameters constrained
Δρ_max_ = 0.30 e Å^−3^
Δρ_min_ = −0.41 e Å^−3^

### 

Data collection: *APEX2* (Bruker, 2005 ▶); cell refinement: *SAINT* (Bruker, 2005 ▶); data reduction: *SAINT*; program(s) used to solve structure: *SHELXS97* (Sheldrick, 2008 ▶); program(s) used to refine structure: *SHELXL97* (Sheldrick, 2008 ▶); molecular graphics: *PLATON* (Spek, 2009 ▶); software used to prepare material for publication: *publCIF* (Westrip, 2010 ▶).

## Supplementary Material

Crystal structure: contains datablock(s) I, global. DOI: 10.1107/S1600536812007519/fj2520sup1.cif

Structure factors: contains datablock(s) I. DOI: 10.1107/S1600536812007519/fj2520Isup2.hkl

Supplementary material file. DOI: 10.1107/S1600536812007519/fj2520Isup3.cml

Additional supplementary materials:  crystallographic information; 3D view; checkCIF report

## Figures and Tables

**Table 1:** Hydrogen-bond geometry (Å, °) *Cg*1 is the centroid of the pyrazole ring.

*D*—H⋯*A*	*D*—H	H⋯*A*	*D*⋯*A*	*D*—H⋯*A*
N1—H1*N*⋯O2^i^	0.88	2.12	2.9779 (15)	164
C3—H3⋯O5^ii^	0.93	2.41	3.3277 (17)	168
C21—H21*B*⋯*Cg*1^iii^	0.93	2.98	3.6660 (18)	130

Symmetry codes: (i) 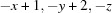; (ii) 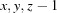; (iii) 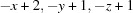.

**Fig. 1.**
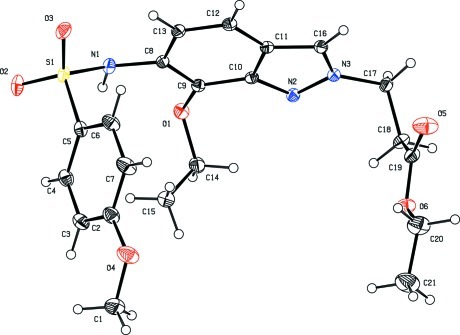
Molecular view of the title compound showing the atom-labeling scheme. Displacement ellipsoids are drawn at the 30% probability level. H atoms are represented as small spheres of arbitrary radii.

**Fig. 2.**
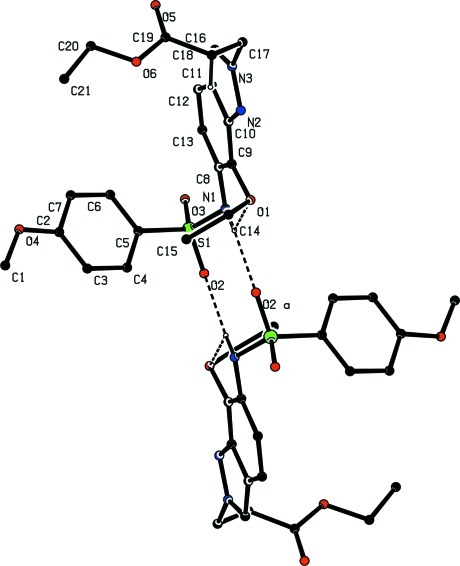
View of the N—H···O bonded dimers of the title compound.

**Fig. 3.**
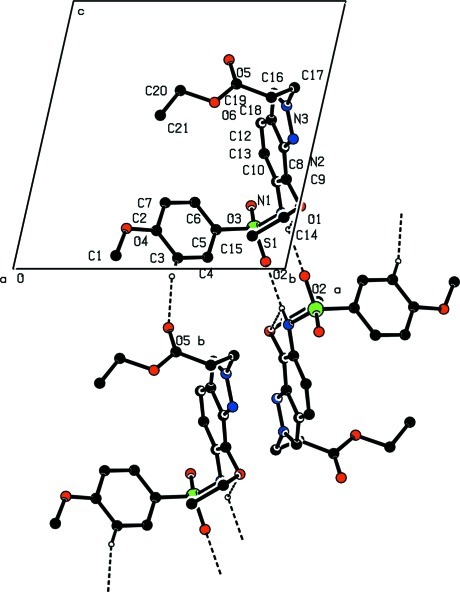
Partial packing view showing N—H···O and C—H···O hydrogen bonds. H atoms not involved in hydrogen bonds have been omitted for clarity.

## supplementary crystallographic information

### Comment

Various sulfonamides are widely used as anti-hypertensive (Soledade *et al.*, 2006; Lee & Lee, 2002). In former papers, we reported the crystal structures of *N*-(7-ethoxy-1*H*-indazol-4-yl)-4-methylbenzenesulfonamide (Abbassi *et al.*, 2011*a*) and *N*-[7-ethoxy-1-(prop-2-en-1-yl)-1*H*-indazol-4-yl]-4-methylbenzenesulfonamide (Abbassi *et al.*, 2011*b*). In this communication, the crystal structure of *N*-[7-ethoxy-2-(prop-2-en-1-yl)-2*H*-indazol-6-yl]-4-methylbenzenesulfonamide is reported.

In the title compound, C_21_H_25_N_3_O_6_S, the dihedral angle between the methoxyphenyl and the indazole rings is: 74.96 (5)° (Fig. 1).

Two neighbouring molecules generate a hydrogen-bonded dimer about a center of inversion through a pair of intermolecular N—H···O interactions (Table 1 and Fig. 2).

The crystal packing is stabilized by intermolecular N—H···O and C—H···O H-bonds and C—H···π interactions (Fig. 3). Also, π–π contacts are observed with centroid-centroid distance of 3.5333 (6) Å.

### Experimental

A mixture of ethyl 3-(6-nitro-2*H*-indazol-2-yl)propanoate (1.22 mmol) and anhydrous SnCl_2_ (1.1 g, 6.1 mmol) in 25 mL of absolute ethanol was heated at 60 °C for 3 h. After reduction, the starting material disappeared, and the solution was allowed to cool down. The pH was made slightly basic (pH 7–8) by addition of 5% aqueous potassium bicarbonate before extraction with ethyl acetate. The organic phase was washed with brine and dried over magnesium sulfate. The solvent was removed to afford the amine, which was immediately dissolved in pyridine (5 ml) and then reacted with 4-methoxybenzenesulfonyl chloride (1.25 mmol) at room temperature for 24 h. After the reaction mixture was concentrated *in vacuo*, the resulting residue was purified by flash chromatography (eluted with Ethyl acetate: Hexane 1:9).

### Refinement

The H atoms bound to C were positioned geometrically and constrained to ride on their parent atoms [C—H distances are 0.93Å for CH groups with *U*_iso_(H) = 1.2 *U*_eq_(C,*N*), and 0.97 Å for CH3 groups, and the coordinates for the H atom bonded to N were taken from a difference map, and the atom was refined using a riding model.

### Crystal data

**Table:**

C_21_H_25_N_3_O_6_S	*Z* = 2
*M**_r_* = 447.50	*F*(000) = 472
Triclinic, *P*1	*D*_x_ = 1.390 Mg m^−^^3^
Hall symbol: -P 1	Mo *K*α radiation, λ = 0.71073 Å
*a* = 9.1163 (4) Å	Cell parameters from 256 reflections
*b* = 10.9161 (5) Å	θ = 1.7–26.3°
*c* = 11.2959 (5) Å	µ = 0.20 mm^−^^1^
α = 77.259 (2)°	*T* = 296 K
β = 77.364 (2)°	Prism, colourless
γ = 88.562 (2)°	0.32 × 0.31 × 0.19 mm
*V* = 1069.55 (8) Å^3^	

### Data collection

**Table:**

Bruker APEXII CCD detector diffractometer	4187 independent reflections
Radiation source: fine-focus sealed tube	3834 reflections with *I* > 2σ(*I*)
Graphite monochromator	*R*_int_ = 0.025
ω and φ scans	θ_max_ = 26.0°, θ_min_ = 2.6°
Absorption correction: multi-scan (*SADABS*; Sheldrick, 2003)	*h* = −11→10
*T*_min_ = 0.940, *T*_max_ = 0.964	*k* = −12→13
21582 measured reflections	*l* = −13→13

### Refinement

**Table:**

Refinement on *F*^2^	Primary atom site location: structure-invariant direct methods
Least-squares matrix: full	Secondary atom site location: difference Fourier map
*R*[*F*^2^ > 2σ(*F*^2^)] = 0.030	Hydrogen site location: inferred from neighbouring sites
*wR*(*F*^2^) = 0.084	H-atom parameters constrained
*S* = 1.06	*w* = 1/[σ^2^(*F*_o_^2^) + (0.0428*P*)^2^ + 0.3561*P*] where *P* = (*F*_o_^2^ + 2*F*_c_^2^)/3
4187 reflections	(Δ/σ)_max_ < 0.001
283 parameters	Δρ_max_ = 0.30 e Å^−^^3^
0 restraints	Δρ_min_ = −0.41 e Å^−^^3^

### Special details

**Table:**

Geometry. All e.s.d.'s (except the e.s.d. in the dihedral angle between two l.s. planes) are estimated using the full covariance matrix. The cell e.s.d.'s are taken into account individually in the estimation of e.s.d.'s in distances, angles and torsion angles; correlations between e.s.d.'s in cell parameters are only used when they are defined by crystal symmetry. An approximate (isotropic) treatment of cell e.s.d.'s is used for estimating e.s.d.'s involving l.s. planes.
Refinement. Refinement of *F*^2^ against ALL reflections. The weighted *R*-factor *wR* and goodness of fit *S* are based on *F*^2^, conventional *R*-factors *R* are based on *F*, with *F* set to zero for negative *F*^2^. The threshold expression of *F*^2^ > σ(*F*^2^) is used only for calculating *R*-factors(gt) *etc*. and is not relevant to the choice of reflections for refinement. *R*-factors based on *F*^2^ are statistically about twice as large as those based on *F*, and *R*- factors based on ALL data will be even larger.

### Fractional atomic coordinates and isotropic or equivalent isotropic displacement parameters (Å^2^)

**Table:**

	*x*	*y*	*z*	*U*_iso_*/*U*_eq_	
S1	0.34987 (3)	0.85372 (3)	0.15120 (3)	0.02632 (10)	
O1	0.72338 (10)	1.00455 (8)	0.23308 (8)	0.0272 (2)	
O2	0.35314 (11)	0.92233 (9)	0.02682 (9)	0.0362 (2)	
O3	0.20739 (10)	0.82306 (10)	0.23512 (9)	0.0349 (2)	
O4	0.67582 (14)	0.38348 (10)	0.15066 (10)	0.0478 (3)	
O5	0.90152 (13)	0.62024 (10)	0.77999 (10)	0.0498 (3)	
O6	1.08922 (11)	0.60258 (9)	0.62130 (9)	0.0351 (2)	
N1	0.44792 (12)	0.94084 (10)	0.20865 (10)	0.0253 (2)	
H1N	0.5206	0.9790	0.1474	0.030*	
N2	0.79639 (11)	0.92171 (9)	0.48444 (9)	0.0228 (2)	
N3	0.76942 (11)	0.87006 (9)	0.60807 (9)	0.0221 (2)	
C1	0.79320 (19)	0.36718 (17)	0.04930 (16)	0.0471 (4)	
H1A	0.7571	0.3869	−0.0263	0.071*	
H1B	0.8247	0.2816	0.0638	0.071*	
H1C	0.8768	0.4221	0.0422	0.071*	
C2	0.60616 (16)	0.49568 (13)	0.14264 (13)	0.0332 (3)	
C3	0.63898 (15)	0.59621 (13)	0.04064 (12)	0.0325 (3)	
H3	0.7137	0.5903	−0.0285	0.039*	
C4	0.55877 (15)	0.70533 (13)	0.04335 (12)	0.0301 (3)	
H4	0.5803	0.7735	−0.0241	0.036*	
C5	0.44676 (14)	0.71363 (12)	0.14587 (11)	0.0253 (3)	
C6	0.41431 (16)	0.61296 (12)	0.24768 (12)	0.0325 (3)	
H6	0.3390	0.6187	0.3164	0.039*	
C7	0.49430 (18)	0.50480 (13)	0.24608 (13)	0.0387 (3)	
H7	0.4737	0.4374	0.3143	0.046*	
C8	0.48957 (13)	0.89671 (11)	0.32512 (11)	0.0220 (2)	
C9	0.63068 (13)	0.92982 (10)	0.33424 (11)	0.0214 (2)	
C10	0.66831 (13)	0.89481 (10)	0.45259 (11)	0.0206 (2)	
C11	0.56294 (13)	0.82540 (10)	0.55620 (11)	0.0213 (2)	
C12	0.42007 (13)	0.79075 (11)	0.54333 (11)	0.0241 (2)	
H12	0.3517	0.7439	0.6106	0.029*	
C13	0.38492 (13)	0.82759 (11)	0.42996 (11)	0.0248 (3)	
H13	0.2903	0.8072	0.4207	0.030*	
C14	0.86846 (14)	0.95273 (14)	0.19168 (12)	0.0322 (3)	
H14A	0.9109	0.9181	0.2633	0.039*	
H14B	0.9358	1.0195	0.1380	0.039*	
C15	0.85789 (17)	0.85207 (15)	0.12261 (14)	0.0389 (3)	
H15A	0.7894	0.7867	0.1747	0.058*	
H15B	0.9554	0.8178	0.1001	0.058*	
H15C	0.8220	0.8872	0.0488	0.058*	
C16	0.63495 (13)	0.81239 (11)	0.65462 (11)	0.0228 (2)	
H16	0.5970	0.7715	0.7368	0.027*	
C17	0.88488 (14)	0.88388 (12)	0.67592 (12)	0.0262 (3)	
H17A	0.8435	0.8573	0.7645	0.031*	
H17B	0.9153	0.9717	0.6585	0.031*	
C18	1.02110 (14)	0.80682 (12)	0.63999 (12)	0.0283 (3)	
H18A	1.0513	0.8232	0.5500	0.034*	
H18B	1.1035	0.8337	0.6707	0.034*	
C19	0.99400 (14)	0.66782 (12)	0.68980 (12)	0.0283 (3)	
C20	1.0816 (2)	0.46675 (14)	0.66528 (16)	0.0480 (4)	
H20A	0.9814	0.4346	0.6710	0.058*	
H20B	1.1051	0.4436	0.7469	0.058*	
C21	1.1945 (2)	0.41394 (17)	0.57294 (18)	0.0606 (5)	
H21A	1.1729	0.4411	0.4919	0.091*	
H21B	1.1894	0.3239	0.5965	0.091*	
H21C	1.2936	0.4431	0.5713	0.091*	

### Atomic displacement parameters (Å^2^)

**Table:**

	*U*^11^	*U*^22^	*U*^33^	*U*^12^	*U*^13^	*U*^23^
S1	0.02522 (17)	0.02943 (17)	0.02551 (17)	0.00073 (12)	−0.01169 (12)	−0.00246 (12)
O1	0.0282 (5)	0.0253 (4)	0.0243 (4)	−0.0030 (4)	−0.0030 (4)	0.0002 (3)
O2	0.0405 (5)	0.0384 (5)	0.0310 (5)	−0.0014 (4)	−0.0197 (4)	0.0021 (4)
O3	0.0230 (5)	0.0450 (6)	0.0374 (5)	0.0014 (4)	−0.0107 (4)	−0.0064 (4)
O4	0.0668 (8)	0.0344 (6)	0.0392 (6)	0.0135 (5)	−0.0037 (5)	−0.0113 (5)
O5	0.0519 (7)	0.0350 (6)	0.0448 (6)	0.0037 (5)	0.0135 (5)	0.0043 (5)
O6	0.0349 (5)	0.0255 (5)	0.0402 (5)	0.0050 (4)	−0.0016 (4)	−0.0043 (4)
N1	0.0270 (5)	0.0244 (5)	0.0248 (5)	0.0009 (4)	−0.0089 (4)	−0.0030 (4)
N2	0.0229 (5)	0.0218 (5)	0.0236 (5)	0.0011 (4)	−0.0054 (4)	−0.0043 (4)
N3	0.0225 (5)	0.0223 (5)	0.0226 (5)	0.0034 (4)	−0.0066 (4)	−0.0060 (4)
C1	0.0470 (9)	0.0505 (9)	0.0501 (9)	0.0125 (7)	−0.0107 (7)	−0.0253 (8)
C2	0.0419 (8)	0.0284 (7)	0.0318 (7)	0.0012 (6)	−0.0092 (6)	−0.0105 (5)
C3	0.0330 (7)	0.0383 (7)	0.0253 (6)	−0.0042 (6)	−0.0017 (5)	−0.0095 (5)
C4	0.0336 (7)	0.0319 (7)	0.0227 (6)	−0.0049 (5)	−0.0053 (5)	−0.0021 (5)
C5	0.0278 (6)	0.0260 (6)	0.0237 (6)	−0.0035 (5)	−0.0088 (5)	−0.0053 (5)
C6	0.0405 (7)	0.0282 (6)	0.0250 (6)	−0.0040 (5)	0.0005 (5)	−0.0052 (5)
C7	0.0571 (9)	0.0256 (7)	0.0275 (7)	−0.0014 (6)	−0.0006 (6)	−0.0021 (5)
C8	0.0248 (6)	0.0201 (5)	0.0228 (6)	0.0059 (4)	−0.0072 (5)	−0.0066 (4)
C9	0.0237 (6)	0.0172 (5)	0.0225 (6)	0.0022 (4)	−0.0033 (5)	−0.0043 (4)
C10	0.0207 (5)	0.0169 (5)	0.0245 (6)	0.0030 (4)	−0.0045 (4)	−0.0059 (4)
C11	0.0217 (6)	0.0188 (5)	0.0232 (6)	0.0035 (4)	−0.0030 (4)	−0.0060 (4)
C12	0.0200 (6)	0.0261 (6)	0.0245 (6)	0.0002 (5)	−0.0009 (4)	−0.0059 (5)
C13	0.0191 (6)	0.0280 (6)	0.0283 (6)	0.0010 (5)	−0.0045 (5)	−0.0091 (5)
C14	0.0234 (6)	0.0442 (8)	0.0260 (6)	−0.0052 (5)	−0.0008 (5)	−0.0051 (6)
C15	0.0371 (8)	0.0470 (8)	0.0340 (7)	0.0094 (6)	−0.0083 (6)	−0.0120 (6)
C16	0.0232 (6)	0.0226 (6)	0.0217 (6)	0.0023 (4)	−0.0029 (4)	−0.0052 (4)
C17	0.0253 (6)	0.0272 (6)	0.0295 (6)	0.0028 (5)	−0.0109 (5)	−0.0087 (5)
C18	0.0218 (6)	0.0274 (6)	0.0342 (7)	0.0008 (5)	−0.0062 (5)	−0.0037 (5)
C19	0.0244 (6)	0.0292 (6)	0.0302 (7)	0.0038 (5)	−0.0073 (5)	−0.0030 (5)
C20	0.0618 (10)	0.0251 (7)	0.0514 (9)	0.0095 (7)	−0.0073 (8)	−0.0026 (6)
C21	0.0853 (14)	0.0385 (9)	0.0568 (11)	0.0231 (9)	−0.0111 (10)	−0.0145 (8)

### Geometric parameters (Å, º)

**Table:**

S1—O3	1.4286 (10)	C7—H7	0.9300
S1—O2	1.4333 (9)	C8—C9	1.3762 (17)
S1—N1	1.6411 (10)	C8—C13	1.4247 (17)
S1—C5	1.7527 (13)	C9—C10	1.4212 (16)
O1—C9	1.3734 (14)	C10—C11	1.4213 (16)
O1—C14	1.4479 (16)	C11—C16	1.3900 (17)
O4—C2	1.3597 (17)	C11—C12	1.4115 (17)
O4—C1	1.4254 (19)	C12—C13	1.3602 (17)
O5—C19	1.1964 (16)	C12—H12	0.9300
O6—C19	1.3332 (16)	C13—H13	0.9300
O6—C20	1.4544 (17)	C14—C15	1.497 (2)
N1—C8	1.4271 (15)	C14—H14A	0.9700
N1—H1N	0.8817	C14—H14B	0.9700
N2—C10	1.3515 (15)	C15—H15A	0.9600
N2—N3	1.3557 (14)	C15—H15B	0.9600
N3—C16	1.3365 (15)	C15—H15C	0.9600
N3—C17	1.4595 (15)	C16—H16	0.9300
C1—H1A	0.9600	C17—C18	1.5139 (17)
C1—H1B	0.9600	C17—H17A	0.9700
C1—H1C	0.9600	C17—H17B	0.9700
C2—C3	1.3889 (19)	C18—C19	1.5038 (18)
C2—C7	1.393 (2)	C18—H18A	0.9700
C3—C4	1.385 (2)	C18—H18B	0.9700
C3—H3	0.9300	C20—C21	1.498 (2)
C4—C5	1.3843 (18)	C20—H20A	0.9700
C4—H4	0.9300	C20—H20B	0.9700
C5—C6	1.3884 (18)	C21—H21A	0.9600
C6—C7	1.373 (2)	C21—H21B	0.9600
C6—H6	0.9300	C21—H21C	0.9600
			
O3—S1—O2	118.61 (6)	C16—C11—C10	104.09 (10)
O3—S1—N1	108.67 (6)	C12—C11—C10	120.73 (11)
O2—S1—N1	105.11 (6)	C13—C12—C11	118.30 (11)
O3—S1—C5	107.80 (6)	C13—C12—H12	120.8
O2—S1—C5	109.22 (6)	C11—C12—H12	120.8
N1—S1—C5	106.87 (6)	C12—C13—C8	121.61 (11)
C9—O1—C14	115.21 (9)	C12—C13—H13	119.2
C2—O4—C1	118.54 (12)	C8—C13—H13	119.2
C19—O6—C20	116.37 (11)	O1—C14—C15	112.18 (11)
C8—N1—S1	122.30 (8)	O1—C14—H14A	109.2
C8—N1—H1N	115.2	C15—C14—H14A	109.2
S1—N1—H1N	107.6	O1—C14—H14B	109.2
C10—N2—N3	103.08 (9)	C15—C14—H14B	109.2
C16—N3—N2	114.45 (10)	H14A—C14—H14B	107.9
C16—N3—C17	127.11 (10)	C14—C15—H15A	109.5
N2—N3—C17	118.43 (10)	C14—C15—H15B	109.5
O4—C1—H1A	109.5	H15A—C15—H15B	109.5
O4—C1—H1B	109.5	C14—C15—H15C	109.5
H1A—C1—H1B	109.5	H15A—C15—H15C	109.5
O4—C1—H1C	109.5	H15B—C15—H15C	109.5
H1A—C1—H1C	109.5	N3—C16—C11	106.58 (10)
H1B—C1—H1C	109.5	N3—C16—H16	126.7
O4—C2—C3	124.55 (13)	C11—C16—H16	126.7
O4—C2—C7	115.06 (12)	N3—C17—C18	111.52 (10)
C3—C2—C7	120.38 (13)	N3—C17—H17A	109.3
C4—C3—C2	119.02 (12)	C18—C17—H17A	109.3
C4—C3—H3	120.5	N3—C17—H17B	109.3
C2—C3—H3	120.5	C18—C17—H17B	109.3
C5—C4—C3	120.42 (12)	H17A—C17—H17B	108.0
C5—C4—H4	119.8	C19—C18—C17	113.44 (10)
C3—C4—H4	119.8	C19—C18—H18A	108.9
C4—C5—C6	120.39 (12)	C17—C18—H18A	108.9
C4—C5—S1	120.29 (10)	C19—C18—H18B	108.9
C6—C5—S1	119.27 (10)	C17—C18—H18B	108.9
C7—C6—C5	119.51 (12)	H18A—C18—H18B	107.7
C7—C6—H6	120.2	O5—C19—O6	123.56 (12)
C5—C6—H6	120.2	O5—C19—C18	125.34 (12)
C6—C7—C2	120.27 (13)	O6—C19—C18	111.09 (11)
C6—C7—H7	119.9	O6—C20—C21	106.89 (13)
C2—C7—H7	119.9	O6—C20—H20A	110.3
C9—C8—C13	121.41 (11)	C21—C20—H20A	110.3
C9—C8—N1	117.62 (11)	O6—C20—H20B	110.3
C13—C8—N1	120.85 (11)	C21—C20—H20B	110.3
O1—C9—C8	119.24 (10)	H20A—C20—H20B	108.6
O1—C9—C10	122.63 (10)	C20—C21—H21A	109.5
C8—C9—C10	117.85 (10)	C20—C21—H21B	109.5
N2—C10—C9	128.11 (11)	H21A—C21—H21B	109.5
N2—C10—C11	111.80 (10)	C20—C21—H21C	109.5
C9—C10—C11	120.07 (11)	H21A—C21—H21C	109.5
C16—C11—C12	135.12 (11)	H21B—C21—H21C	109.5
			
O3—S1—N1—C8	61.31 (11)	N1—C8—C9—C10	175.02 (10)
O2—S1—N1—C8	−170.75 (9)	N3—N2—C10—C9	177.71 (11)
C5—S1—N1—C8	−54.76 (11)	N3—N2—C10—C11	−0.64 (12)
C10—N2—N3—C16	0.57 (13)	O1—C9—C10—N2	−3.11 (18)
C10—N2—N3—C17	−178.49 (10)	C8—C9—C10—N2	−176.86 (11)
C1—O4—C2—C3	1.4 (2)	O1—C9—C10—C11	175.13 (10)
C1—O4—C2—C7	−179.11 (13)	C8—C9—C10—C11	1.37 (16)
O4—C2—C3—C4	179.34 (13)	N2—C10—C11—C16	0.50 (13)
C7—C2—C3—C4	−0.1 (2)	C9—C10—C11—C16	−178.00 (10)
C2—C3—C4—C5	−0.5 (2)	N2—C10—C11—C12	178.30 (10)
C3—C4—C5—C6	0.5 (2)	C9—C10—C11—C12	−0.20 (16)
C3—C4—C5—S1	177.81 (10)	C16—C11—C12—C13	175.71 (13)
O3—S1—C5—C4	156.16 (10)	C10—C11—C12—C13	−1.27 (17)
O2—S1—C5—C4	26.04 (12)	C11—C12—C13—C8	1.56 (18)
N1—S1—C5—C4	−87.18 (11)	C9—C8—C13—C12	−0.36 (18)
O3—S1—C5—C6	−26.52 (12)	N1—C8—C13—C12	−176.38 (11)
O2—S1—C5—C6	−156.64 (10)	C9—O1—C14—C15	75.69 (14)
N1—S1—C5—C6	90.14 (11)	N2—N3—C16—C11	−0.27 (13)
C4—C5—C6—C7	0.0 (2)	C17—N3—C16—C11	178.69 (10)
S1—C5—C6—C7	−177.28 (11)	C12—C11—C16—N3	−177.46 (13)
C5—C6—C7—C2	−0.6 (2)	C10—C11—C16—N3	−0.14 (12)
O4—C2—C7—C6	−178.83 (13)	C16—N3—C17—C18	111.14 (13)
C3—C2—C7—C6	0.6 (2)	N2—N3—C17—C18	−69.93 (13)
S1—N1—C8—C9	141.07 (10)	N3—C17—C18—C19	−72.33 (14)
S1—N1—C8—C13	−42.76 (15)	C20—O6—C19—O5	−3.1 (2)
C14—O1—C9—C8	−123.84 (12)	C20—O6—C19—C18	175.71 (12)
C14—O1—C9—C10	62.49 (14)	C17—C18—C19—O5	−24.91 (19)
C13—C8—C9—O1	−175.09 (10)	C17—C18—C19—O6	156.26 (11)
N1—C8—C9—O1	1.05 (16)	C19—O6—C20—C21	178.54 (14)
C13—C8—C9—C10	−1.12 (17)		

### Hydrogen-bond geometry (Å, º)

Cg1 is the centroid of the pyrazole ring.

**Table:**

*D*—H···*A*	*D*—H	H···*A*	*D*···*A*	*D*—H···*A*
N1—H1*N*···O2^i^	0.88	2.12	2.9779 (15)	164
C3—H3···O5^ii^	0.93	2.41	3.3277 (17)	168
C21—H21*B*···*Cg*1^iii^	0.93	2.98	3.6660 (18)	130

Symmetry codes: (i) −*x*+1, −*y*+2, −*z*; (ii) *x*, *y*, *z*−1; (iii) −*x*+2, −*y*+1, −*z*+1.

## References

[bb1] Abbassi, N., Rakib, E. M. & Zouihri, H. (2011*a*). *Acta Cryst.* E**67**, o1354. 10.1107/S1600536811016576 PMC3120551 21754748

[bb2] Abbassi, N., Rakib, E. M. & Zouihri, H. (2011*b*). *Acta Cryst.* E**67**, o1561. 10.1107/S1600536811019465 PMC3151999 21836974

[bb3] Bruker (2005). *APEX2* and *SAINT* Bruker AXS Inc., Madison, Wisconsin, USA.

[bb4] Lee, J. S. & Lee, C. H. (2002). *Bull. Korean Chem. Soc.* **23**, 167–169.

[bb5] Sheldrick, G. M. (2003). *SADABS* University of Göttingen, Germany.

[bb6] Sheldrick, G. M. (2008). *Acta Cryst.* A**64**, 112–122. 10.1107/S0108767307043930 18156677

[bb7] Soledade, M., Pedras, C. & Jha, M. (2006). *Bioorg. Med. Chem.* **14**, 4958–4979. 10.1016/j.bmc.2006.03.014 16616505

[bb8] Spek, A. L. (2009). *Acta Cryst.* D**65**, 148–155. 10.1107/S090744490804362X PMC2631630 19171970

[bb9] Westrip, S. P. (2010). *J. Appl. Cryst.* **43**, 920–925.

